# An Efficient CNN-Based Method for Intracranial Hemorrhage Segmentation from Computerized Tomography Imaging

**DOI:** 10.3390/jimaging10040077

**Published:** 2024-03-25

**Authors:** Quoc Tuan Hoang, Xuan Hien Pham, Xuan Thang Trinh, Anh Vu Le, Minh V. Bui, Trung Thanh Bui

**Affiliations:** 1 Faculty of Mechanical Engineering, Hung Yen University of Technology and Education, 39Rd., Hung Yen 160000, Vietnam; hoangquoctuan@utehy.edu.vn (Q.T.H.); xttrinh@utehy.edu.vn (X.T.T.); 2 Faculty of Mechanical Engineering, University of Transport and Communications, Hanoi 100000, Vietnam; hienpx@utc.edu.vn; 3 Communication and Signal Processing Research Group, Faculty of Electrical and Electronics Engineering, Ton Duc Thang University, Ho Chi Minh City 700000, Vietnam; 4 Faculty of Engineering and Technology, Nguyen Tat Thanh University, 300A, Nguyen Tat Thanh, Ward 13, District 4, Ho Chi Minh City 700000, Vietnam; bvminh@ntt.edu.vn

**Keywords:** computer-aided diagnosis, computer-aided diagnosis, CT scans, convolutional network, data augmentation, intracranial hemorrhage

## Abstract

Intracranial hemorrhage (ICH) resulting from traumatic brain injury is a serious issue, often leading to death or long-term disability if not promptly diagnosed. Currently, doctors primarily use Computerized Tomography (CT) scans to detect and precisely locate a hemorrhage, typically interpreted by radiologists. However, this diagnostic process heavily relies on the expertise of medical professionals. To address potential errors, computer-aided diagnosis systems have been developed. In this study, we propose a new method that enhances the localization and segmentation of ICH lesions in CT scans by using multiple images created through different data augmentation techniques. We integrate residual connections into a U-Net-based segmentation network to improve the training efficiency. Our experiments, based on 82 CT scans from traumatic brain injury patients, validate the effectiveness of our approach, achieving an IOU score of 0.807 ± 0.03 for ICH segmentation using 10-fold cross-validation.

## 1. Introduction

### 1.1. Introduction to Traumatic Brain Injury

Traumatic brain injury (TBI), commonly referred to as head trauma, encompasses injuries to the skull and brain resulting from sudden impacts or penetrating injuries. It can occur due to various incidents such as accidents, falls, assaults, or sports-related activities. These injuries can lead to severe consequences, ranging from superficial wounds to deep internal damage caused by specific objects such as bullets, knives, or bone fragments [1].

TBI varies depending on factors such as the location of the injury, the force of the impact, and the severity of the trauma. The resulting sequelae can include intracranial hematoma, cerebral edema, intracranial hypertension syndrome, brain herniation, and cerebral ischemia [2]. While the cerebral cortex is often the primary site of injury, traumatic incidents can affect other areas of the brain as well. One of the most critical risks is the potential rupture of blood vessels within the brain, leading to the accumulation of blood within the skull. These intracranial hematomas can occur in various regions of the brain, including the subdural, epidural, and intracerebral spaces. Without prompt medical intervention, hematomas can lead to complications such as brain hemorrhage, particularly if they impede blood circulation and result in overflow into the brain’s ventricles [3]. Different locations of intracranial hematomas pose varying degrees of danger, depending on their impact on neurological function and the extent of brain tissue compression.

Cerebral edema, a common consequence of traumatic brain injury, presents significant risks to patients as it appears in its two main forms: cytotoxic edema, resulting from cellular damage, and vasogenic edema, caused by disruptions in vascular integrity. Both types of edemas pose life-threatening dangers [4]. Intracranial hypertension syndrome often accompanies cerebral edema following traumatic brain injury, further endangering patients’ lives [5]. Brain herniation, another sequela of severe cerebral edema, occurs when increased pressure forces the brain tissue through openings or cavities in the skull. This compression, particularly of vital areas like the medulla oblongata, can swiftly lead to fatal outcomes if left untreated [6].

Traumatic brain injury damages the brain tissue, causing swelling, inflammation, and bleeding in the brain and compressing the cerebral blood vessels. When the blood supply does not reach all the brain cells, ischemia will appear in some areas. If the brain is deprived of blood for too long, brain tissue can become irreversibly necrotic. Milder cases of cerebral ischemia are still reversible if the intervention ensures adequate perfusion [7].

### 1.2. Diagnostic Approaches to Traumatic Brain Injury

Advancements in science and technology have revolutionized the diagnosis of traumatic brain injury. Various imaging techniques, such as X-ray, ultrasound, computed tomography (CT), and magnetic resonance imaging (MRI), enable healthcare professionals to visualize and assess brain injuries with precision and accuracy [8,9,10].

X-ray imaging is widely employed in medical facilities to diagnose traumatic brain injury and assess the extent of injuries. It provides valuable insights into the underlying causes and helps clinicians monitor disease progression and treatment efficacy [9]. Additionally, ultrasound imaging offers a non-invasive approach to visualizing soft tissues and organs, making it particularly useful in diagnosing cardiovascular, thyroid, and breast conditions, as well as monitoring pregnancies [10]. Computed tomography (CT) has emerged as a cornerstone in the diagnosis of traumatic brain injury, offering detailed cross-sectional images of the brain and surrounding structures. Its rapid acquisition time and high sensitivity make it indispensable in emergency settings, where timely diagnosis is paramount [8,11]. Furthermore, magnetic resonance imaging (MRI) provides unparalleled detail and clarity in visualizing brain injuries, making it a valuable tool in assessing the extent and severity of traumatic brain injury [8,11].

Despite the significant benefits of imaging techniques in diagnosing traumatic brain injury, challenges remain in ensuring consistent diagnostic performance across healthcare settings. Reliance on individual clinician expertise and experience can lead to variations in diagnostic accuracy and may impact patient outcomes. To address these challenges, computer-aided diagnosis (CAD) systems have been developed to support healthcare professionals in interpreting medical images and improving the diagnostic accuracy. CAD systems leverage advanced algorithms to analyze medical images and provide diagnostic insights to clinicians. By integrating rich datasets and leveraging machine learning techniques, CAD systems can enhance the diagnostic efficiency and reduce the impact of individual clinicians’ variability. However, the development and implementation of CAD systems require rigorous validation and continuous refinement to ensure optimal performance and clinical utility.

Computerized Tomography (CT) is a rapid imaging technique known for its high sensitivity in identifying traumatic brain injury. It is widely regarded the gold standard for diagnosing TBI in emergency departments due to its widespread availability. Radiologists examine CT images to identify intracranial hemorrhage (ICH) and assess its type, location, and extent. However, this diagnostic process relies heavily on the expertise of highly trained physicians, leading to potential limitations in diagnosis time and accuracy, particularly in underserved areas lacking specialized care services and experienced radiologists [12].

### 1.3. Advanced Imaging Techniques and the Role of Deep Learning

In recent years, deep learning algorithms, particularly convolutional neural networks (CNNs), have shown great promise in automating the detection and segmentation of intracranial hemorrhage in CT scans [13]. Previous studies have demonstrated the effectiveness of CNNs in detecting and segmenting intracranial hemorrhage. For example, W. Kuo et al. [14] trained a fully convolutional neural network to localize abnormalities in head CT scans, while Y. Yuan et al. [15] utilized a CNN based on the U-Net structure for segmenting irregular cerebral hemorrhage images. These advancements in deep learning hold the potential to enhance the efficiency and accuracy of TBI diagnosis, offering valuable support to healthcare professionals in their clinical practice.

In this study, we propose a novel approach to segmenting intracranial hemorrhage regions in CT scans using a partitioning network based on an automatic encoder–decoder architecture [16]. By leveraging the capabilities of deep learning and incorporating residual connections, we aim to develop an automated model for detecting and segmenting intracranial hemorrhage, thereby enhancing the diagnostic capabilities and facilitating timely intervention in cases of traumatic brain injury. Computer-aided diagnosis systems have the potential to revolutionize the diagnosis and management of traumatic brain injury by providing clinicians with valuable insights and decision support tools. By leveraging the power of advanced algorithms and machine learning techniques, CAD systems can augment clinician expertise, improve diagnostic accuracy, and ultimately enhance patient outcomes [17].

## 2. Proposed Method

### 2.1. Overview of the Proposed Method

In the training of a deep learning (DL) model, the dataset is typically split into training and testing subsets. During the training stage, the training dataset is passed through the DL network over multiple iterations (referred to as epochs), optimizing the network parameters to enhance the desired outcomes in each epoch. Subsequently, the model’s performance is assessed using the test data from the original dataset.

Overfitting is a significant concern in deep learning, especially when dealing with limited training data, as it can lead to reduced accuracy in tasks such as image segmentation. In this study, we address this challenge using two main strategies. Firstly, we employ data augmentation techniques to expand the training dataset artificially. Secondly, we propose a partitioning network based on a U-Net architecture, augmented with additional sub-association blocks to enhance the partitioning accuracy compared to prior methodologies. To validate our approach, we conduct experiments using a publicly available dataset.

**Figure 1:**
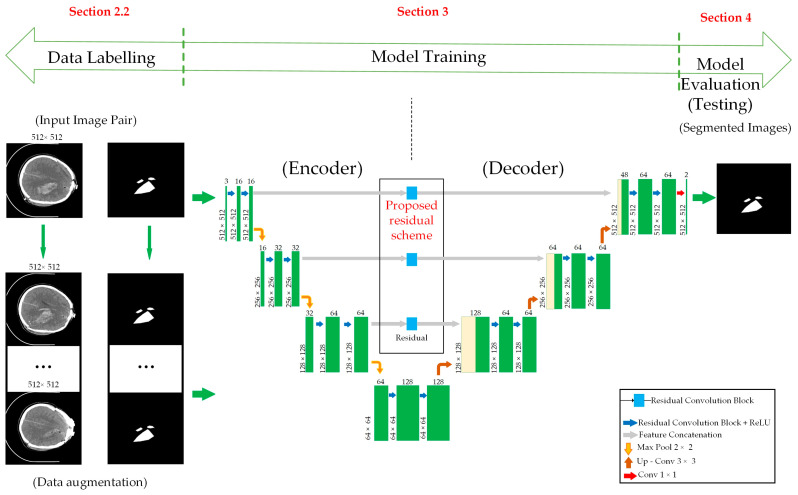
Overall flow chart of the proposed method.

A schematic representation of our proposed approach is outlined in Figure 1.

The proposed method comprises two essential steps. Initially, standard data augmentation techniques are employed on the original dataset images, as described in Section 2.2, resulting in the creation of a new dataset, as outlined in Section 2.4. This initial step effectively expands the training dataset compared to its original size. Subsequently, a unified segmentation network is designed based on the foundation of the conventional U-Net architecture. This network incorporates supplementary auxiliary skip connections, facilitating the construction of a deeper segmentation network without compromising the training process, thereby bolstering the accuracy of detection and segmentation, as discussed in Section 2.3. Finally, we utilize established metrics to evaluate the performance of the proposed partition model, which are detailed in Section 3 of the paper.

### 2.2. Dataset

Intracranial hemorrhages (ICHs) pose significant risks, potentially leading to paralysis or fatality without prompt and accurate diagnosis followed by emergency intervention. Currently, computed tomography (CT) scans are the primary diagnostic tool utilized by medical professionals to find the precise location of such lesions. A study was conducted on individuals suffering from traumatic brain injuries, with a focus on collecting and analyzing their skull CT scans [18].

**Figure 2:**
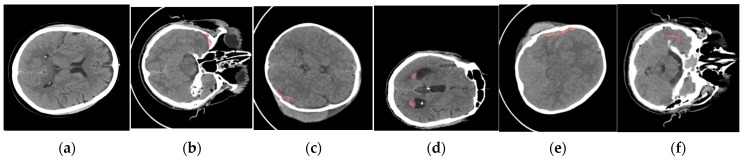
Dataset of intracranial hemorrhage images: (**a**) no ICH, (**b**) epidural, (**c**) intraparenchymal, (**d**) intraventricular, (**e**) subdural, and (**f**) subarachnoid. The area inside the red line indicated the area of ICH [18].

The dataset comprises CT scan images obtained from 82 patients with traumatic brain injuries, collected in 2018 at Al Hilla Teaching Hospital in Iraq [18]. Among these scans, 36 were identified as having intracerebral hemorrhages, categorized into five distinct types, intraventricular hemorrhage, intraparenchymal hemorrhage, subarachnoid hemorrhage, epidural hemorrhage, and subdural hemorrhage, as illustrated in Figure 2.

Each patient’s brain CT scan typically consisted of approximately 30 slices, with each slice having a thickness of 5 mm. Among the 82 patients included in the study (46 men and 36 women), with an average age of 27.8 ± 19.5 years, a thorough examination was conducted by a medical professional to classify each slice for the presence of a hemorrhage or skull fracture [18].

Subsequently, the radiologist’s annotations were meticulously localized using a custom tool in MATLAB. This tool precisely identified any hemorrhages and represented them as white areas against a black background in the CT scan images. These annotated images, known as mask images, were then paired with their corresponding CT scans.

**Figure 3:**
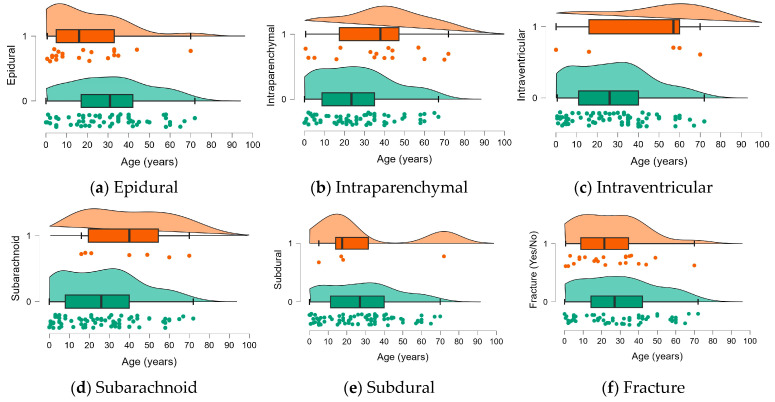
Demographic and clinical characteristics of the original dataset [18].

Finally, both the original CT scans and their associated mask images were saved as NIfTI files, resulting in a total of 75 NIfTI files, comprising 75 skull CT images and their corresponding mask image files. The dataset structure includes the following folders and files is presented in Table 1:

In Figure 3, the demographic and clinical characteristics of the original dataset are described as raincloud plots with jittering and the median, IQR boxes, and CI95 intervals visualized for each quantitative characteristic, like age, between the control and affected samples.

### 2.3. Deep-Learning-Based Intracranial Hemorrhage Segmentation

In this study, we have developed an intracranial hemorrhage segmentation network based on an autoencoder–decoder architecture. Unlike traditional convolutional neural networks (CNNs), our segmentation network excludes a fully connected layer. The encoder component of the segmentation network serves to extract features from input images, similar to the convolutional layers in CNNs. Conversely, the decoder component utilizes these extracted features to reconstruct images containing the segmented object.

**Figure 4:**
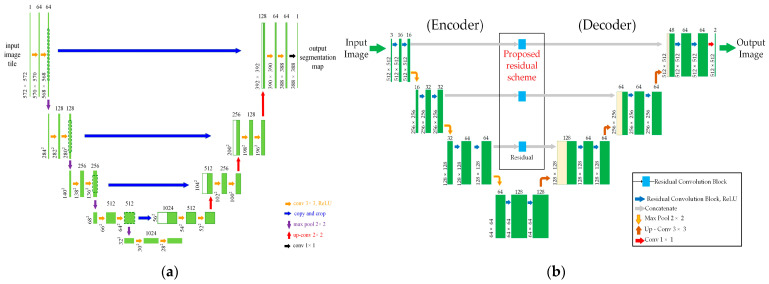
Architecture of the segment network: (**a**) standard U-Net architecture [19], (**b**) architecture of the proposed segment network.

Within the domain of segmentation networks, three prominent architectures have emerged: U-Net [19], ResNet50′s application [20], and fully convolutional networks (FCNs) [21], as shown in Figure 4a. Inspired by the concept of residual connections, we have tailored a segmentation network specifically to intracranial hemorrhage area detection in CT images, adopting a structure akin to that of U-Net.

Our network architecture consists of two primary segments: the contraction (or encoder) branch on the left and the expansion (or decoder) branch on the right.

Encoder: This section mirrors a conventional convolutional neural network (CNN) and is responsible for feature extraction. The layer dimensions progressively decrease from an initial size of 512 × 512 to 64 × 64, while the depth of the network gradually increases from 3 to 128.

Decoder: Symmetrically aligned with the encoder branch, the decoder section employs upsampling techniques to gradually restore the dimensions of the layers. Ultimately, the segmentation network produces a mask image, indicating the predicted label for each pixel.

Our network structure closely resembles that of a standard U-Net architecture, as illustrated in Figure 4a, with the key distinction being the integration of residual connections for image information transmission, as opposed to conventional convolutional layers, as illustrated in Figure 4b.

**Figure 5:**
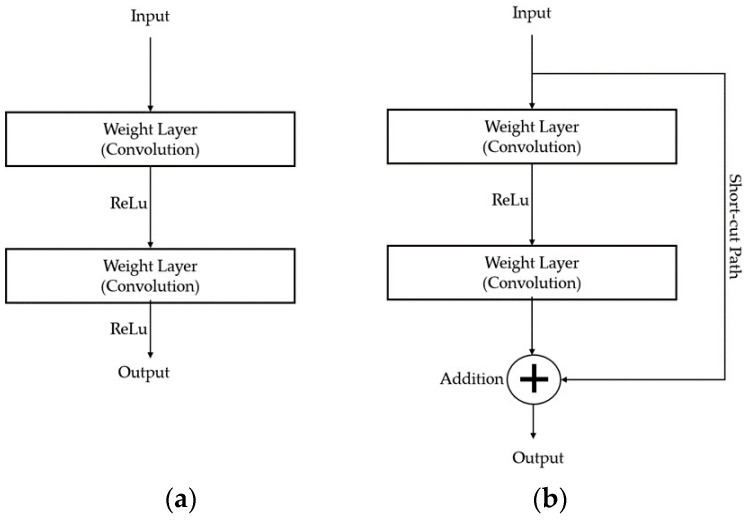
Diagram of conventional convolution blocks (**a**,**b**). Residual block used in deep learning networks [22].

Furthermore, we enhance the segmentation network by incorporating residual blocks, as shown in Figure 5b [22], instead of traditional convolutional layers, as illustrated in Figure 5a, in the reduced paths. By combining two feature matrices, as depicted in Figure 5b, the output of the residual block synthesizes the shallow and deep layers to obtain special fused features. Layers at the same level are aggregated and included in the previous layer. Additionally, features from the shallower block of the network are combined with the final feature map in a skip-connect fashion to avoid any dimensionality reduction [23]. The synthesis of deep features across layers with the proposed method is expressed using Equation (1) as follows:

(1) $$F_{d}=A\left(\left(R_{d-1}^{d}\left(x\right),R_{d-2}^{d}\left(x\right),\ldots ,R_{1}^{n}\left(x\right),L_{1}^{n}\left(x\right),L_{2}^{n}\left(x\right)\right)\right).$$

In Equation (1), *F* represents the final feature map, whereas *d*, *A*, and *x* represent the depth, aggregation node, and feature map at level x, respectively. *L* and *R* in Equation (1) are defined in Equations (2) and (3) as follows:

(2) $$L_{2}^{d}\left(x\right)=C\left(L_{1}^{d}\left(x\right)\right),L_{1}^{d}\left(x\right)=C\left(R_{1}^{d}\left(x\right)\right),$$

(3) $$R_{m}^{d}\left(x\right)=\left\{\begin{array}{l} F_{m}\left(x\right),if\;m=n-1\\ F_{m}\left(R_{m+1}^{d}\left(x\right)\right),otherwise \end{array}\right..$$

This alteration has been demonstrated to improve the network depth and learning capacity and mitigate issues such as gradient vanishing. Each residual connection layer comprises three convolutional layers, resulting in a greater number of layers compared to the traditional U-Net model [19]. At the final layer of the segmentation network, based on the U-Net architecture, we apply the sigmoid activation function to predict the probability of each label.

### 2.4. Data Augmentation

The application of deep learning to medical image analysis often encounters the problem of the limited availability of labeled training data, which can be both costly and time-consuming to obtain. To address this issue, data augmentation techniques are frequently employed to augment the size and diversity of the training dataset. This serves as a pivotal strategy to alleviate overfitting and enhance the model performance. As detailed in the preceding section, our dataset comprises 75 NIfTI images extracted from brain CT scans, of which only 36 cases were diagnosed with intracerebral hemorrhage. Post-execution, we identified 318 slices exhibiting hemorrhages among the 36 NIfTI files, with each slice represented as a 512 × 512 grayscale image categorized into five distinct types of intracerebral hemorrhage.

Recognizing the relatively small dataset size, we undertook data augmentation to expand our training dataset. Additionally, we observed significant disparities in the distribution of images across the five hemorrhage types, as depicted in Table 1. Consequently, we decided not to divide the dataset into 5 types and aggregated these data together into a single category, namely intracerebral hemorrhage.

Subsequently, we applied three data augmentation methods: image flipping, brightness adjustment, and rotation. Initially, the images were horizontally and vertically flipped, with corresponding adjustments made to the mask images. Brightness alterations were then introduced, with a 10% decrease applied to vertically flipped images and a 10% increase to their counterparts. Since the brightness adjustments did not affect the hemorrhage position, no modifications were made to the mask images at this stage. Subsequent rotation involved a 10° counterclockwise rotation followed by a 5° rotation, centered on the image’s axis. Corresponding adjustments were made to the mask images.

Given the varying quantities of images across different hemorrhage types, the augmentation solutions were customized to address the dataset imbalances. Images containing unclear circular hemorrhage areas underwent brightness adjustment, while slight rotations of 5 or 10 degrees were applied to generate additional images in cases where patients had tilted their heads during CT scans, thereby enriching the diversity of the training dataset. Additionally, all six augmentation solutions outlined in Table 2 were applied to address limited data cases, namely intraventricular and subarachnoid hemorrhages. Data augmentation was implemented using the OpenCV library with Python in the PyCharm environment.

Following the data augmentation, the five hemorrhage types were merged into a single category, overwriting duplicate images where applicable. The image dimensions remained at 512 × 512 to facilitate the U-Net training.

After the data augmentation was implemented, the initial set of 318 slice images expanded to a dataset comprising 832 slice images, as detailed in Table 2. This augmented dataset demonstrated a more balanced distribution of slice images across different hemorrhage types, thereby enhancing the efficacy of the subsequent training and testing processes for the proposed segmentation network. Only the cross-validation strategy was used in this study, without testing on the special testing group.

**Figure 6:**
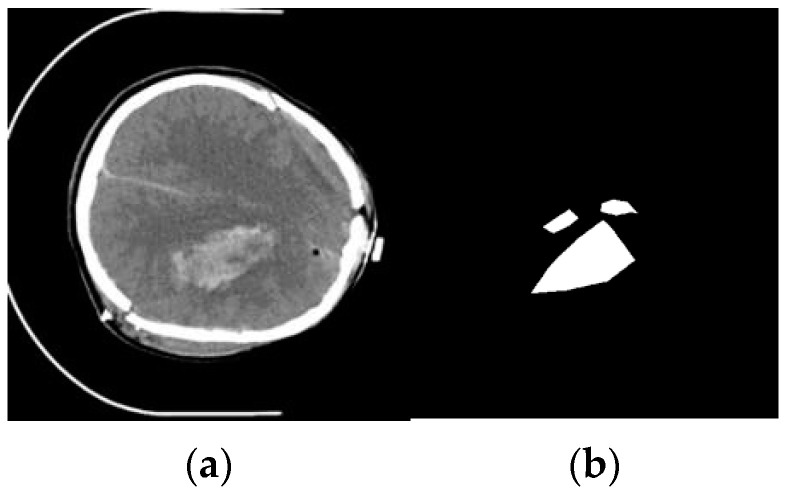
Example image processing for training: (**a**) slice and (**b**) mass image of slice with hemorrhage area pixels of 255 (white).

Notably, in the mask images, background pixels were represented with a value of 0 (black), while pixels corresponding to the hemorrhage areas marked by medical professionals had a value of 255 (white). To facilitate training, we standardized the mask images by converting the hemorrhage area pixels with a value of 255 into 1. In summary, the mask images utilized for training were binarized to include two distinct pixel values, 0 for the background areas and 1 for the hemorrhage areas, as illustrated in Figure 6.

## 3. Experimental Results

### 3.1. Performance Measurement Metrics

To assess the effectiveness of the proposed model, we utilized the Jaccard index, also known as the Intersection over the Union (*IOU*) score index. The optimal model achieves IOU scores close to 1, indicating high accuracy. If the *IOU* measurement result exceeds or equals 0.5, it signifies a true positive (*TP*) case; otherwise, it is considered a false positive (*FP*) case. False negative (*FN*) cases occur when the system fails to detect any object from the input image with the ground truth object. The *IOU* score index is computed using the following equation:

(4) $$IOU=Jaccard(A,B)=\frac{A\cap B}{A\cup B}$$

As segmentation methods can be treated as detection problems, we employed three metrics, precision (Equation (5)), recall (Equation (6)), and the F1-score (Equation (7)), as established in previous studies [24]. Additionally, for the segmentation task, we utilized overall accuracy (Equation (8)) and specificity (Equation (9)) to evaluate the performance.

(5) $$Precision=\frac{TP}{TP+FP}$$

(6) $$Recall=\frac{TP}{TP+FN}$$

(7) $$F_{1}=\frac{2*(Recall*Precision)}{Recall+Precision}$$

(8) $$Accuracy=\frac{TP+TN}{TP+FP+TN+FN}$$

(9) $$Specificity=\frac{TN}{TN+FP}$$

In these equations, *TP*, *FP*, *TN*, and *FN* represent true positives, false positives, true negatives, and false negatives, respectively, as per the standard confusion matrix.

### 3.2. Experimental Results

This section outlines the experimental methodology employed in our study. We utilized the standard K-fold cross-validation technique by setting K to 10 to evaluate the detection model. We opted for 10-fold cross-validation as it aligns with references [25,26], which suggest that K should be chosen to ensure the training data exhibit sufficient variance for effective learning. The value for K is fixed at 10, a choice validated through experimentation, typically resulting in a model skill estimate with low bias and modest variance. Decreasing K increases the bias in the estimate. This occurs because, with lower values of K, the model trains on fewer data, leading to a pessimistic bias in the estimate as the number of data available for learning decreases. Conversely, as K increases, the difference in size between the training set and the resampling subsets diminishes. Consequently, the bias of the technique diminishes as well.

In this method, labeled images were divided into ten subsets, with nine sets designated for training the detection network and one set reserved for evaluation. This iterative process was repeated ten times to ensure comprehensive utilization of the data for training and testing, thereby reducing bias in the model performance assessment. This approach was vital for eliminating the biases associated with the specific dataset splits for training and testing. During each cross-validation iteration, a new model was trained independently. Subsequently, the trained model was validated using the test dataset, and the results were recorded for each validation. This process was repeated ten times, and the final outcome was determined by averaging the results across all validations.

The obtained experimental results from the detection model demonstrated reasonable accuracy. The deep learning models were implemented and were trained on hardware equipped with an Intel Core i7-8700k, 64 GB of RAM, and NVIDIA GeForce GTX 1080 Ti graphics cards.

We implemented the U-Net architecture, illustrated in Figure 4, with Python 3.9, using the Keras library with TensorFlow support [27,28]. The network backbone is the Resnet34 network [29]. The dimensions of the input images were 512 × 512. During the experiment, we used the Adam optimizer algorithm with a learning rate of 0.001 and a batch size of 16.

**Figure 7:**
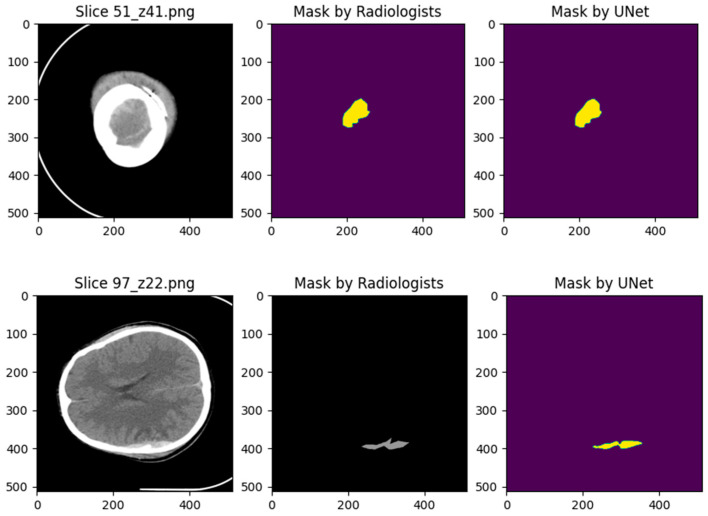
The results of the proposed model are presented using Matplotlib.

The results are presented visually using the Matplotlib library, as shown in Figure 7. where A is the bleeding area indicated by the doctors and B is the bleeding area indicated by the model.

After conducting training and testing with the test dataset, the result achieved an IOU score of 0.808. This indicated that the IOU score obtained using the proposed method was close to 1. It means that the results obtained in this study are highly satisfactory.

To assess the effectiveness of our proposed approach, an experiment was conducted to compare its performance with that reported in prior studies. This experiment specifically focused on comparing the segmentation performance across datasets [18], as outlined in Table 3 and Table 4. Zhou et al. [30] recently introduced a novel network architecture named the UNet++ network, which has demonstrated efficacy in addressing medical image segmentation challenges. The UNet++ network can be viewed as a nested structure comprising both shallow and deep U-Net networks, resulting in improved segmentation performance compared to conventional U-Net networks. To facilitate a comprehensive comparison, we conducted experiments utilizing the UNet++ network on the datasets, as detailed in the following sections.

**Figure 8:**
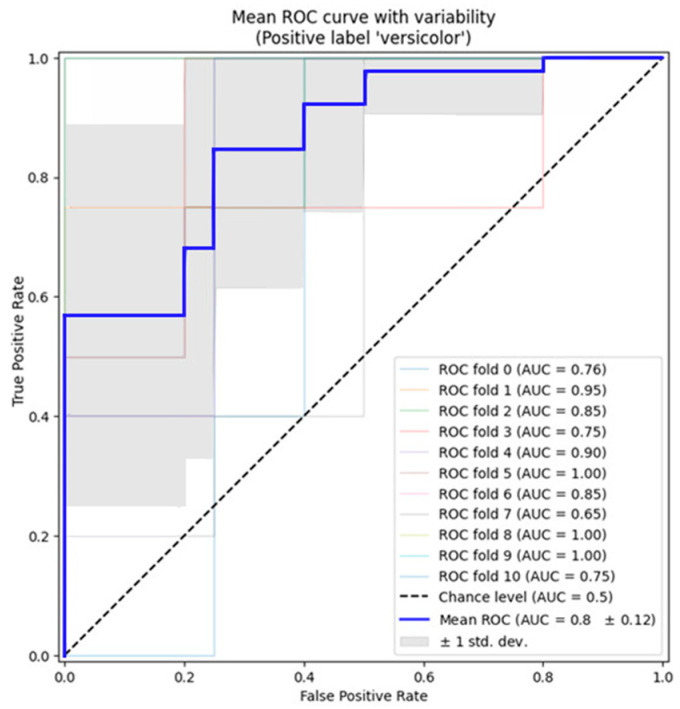
The results of the cross-validation procedure in a form of AUC ROC curves.

In Table 3, we provide a comparative analysis of the proposed approach’s performance when utilized as an object detection framework against other solutions. Following, in Table 4, we present the results of employing the proposed approach as a segmentation framework and compare them with other segmentation frameworks to show the considerable outperform outcomes in the IOU-0.9, specificity, and accuracy criteria. We run the classifier with cross-validation and plot the ROC curves fold-wise, as shown in Figure 8, with the uncertainty boundaries for all the evaluation indicators reported in the Table 3. Notice that the baseline for defining the chance level (dashed ROC curve) is a classifier that will always predict the most frequent class.

We executed the classifier with cross-validation and generated ROC curves for each fold, including the uncertainty boundaries for all the evaluation indicators, as detailed in Table 3. It is important to note that the dashed ROC curve represents the baseline, which is a classifier predicting the most frequent class consistently. By considering all these curves, we computed the mean Area Under the Curve (AUC) and depicted it in Figure 8, allowing us to observe the variance in the curve as the training set is partitioned into different subsets. The resulting data provide insights into how the classifier’s output is influenced by variations in the training data and the extent of dissimilarity between the splits generated using 10-fold cross-validation.

The demographic and clinical characteristics of the original dataset were statistically tested to identify potential differences between groups. The results of this testing, depicted in Figure 3, are reported in Table 5 of this paper. The findings indicate that the proposed method successfully detected and segmented data from a total of 82 subjects, with 46 males and an average age of 27.8 ± 19.5 years (refer to Figure 3 for subject demographics). Each CT scan comprises approximately 34 slices on average. Notably, one subject exhibited a small intraparenchymal hemorrhage (IPH) region in a single CT slice, while another subject had a small IPH region in two CT slices. The dimensions of the IPH regions in these cases were below 10 mm in width and height, establishing the lower limit for ICH segmentation achievable using the proposed architecture. Regarding age-related analysis, the accuracy coefficient for subjects under 18 years old was 96.76%, while it was 95.63% for subjects aged 18 and above. Similarly, the accuracy coefficient for male subjects was 95.26%, while it was 96.35% for female subjects. The output indicates that there is no significant difference in the method’s performance between subjects younger and older than 18 years old, as well as between male and female subjects.

## 4. Discussion

As shown in Section 3 and Section 4, the solution implemented in this study adeptly accomplishes the segmentation of the areas in the CT images. Figure 5 provides a visual representation of the experimental results, showcasing the efficacy of the implemented solution using test images derived from the dataset elucidated in Section 2.2. The experimental outcomes substantiate that the segmentation system, employing the proposed approach in this study, proficiently separates intracerebral hemorrhages (ICHs) from CT images. Visually, there exists a notable resemblance between the results obtained by the image experts and those generated by the proposed segmentation system.

To evaluate the effectiveness of the experimental results, the Intersection over the Union (IOU) index, specificity, and accuracy was employed, yielding commendable results of 0.8075, 98.17, and 96.06, respectively. This surpasses the reported result of 0.218 in the prior study by M. Hussain and colleagues, signifying the superior performance of our proposed method. Furthermore, an extensive evaluation of our model was conducted in terms of recall, precision, and the F1-score, encompassing various segmentation networks, including a fully connected network with Residual U-Net [19], FCN-AlexNet [21], FRCNN [21] and UNet++ [30].

Utilizing FCN-AlexNet, we achieved a recall of 90.81%, a precision of 86.03%, and an F1-score of 88.35%. Similarly, FRCNN demonstrated recall, precision, and F1-score values of 91.40%, 93.72%, and 92.56%, respectively. The implementation of Residual U-Net yielded remarkable results with a recall of 98.75%, a precision of 94.31%, and an F1-score of 96.42%. UNet++ exhibited improved performance with a recall of 98.69%, a precision of 92.51%, and an F1-score of 95.43%. In a comparative analysis, our proposed method outshines the aforementioned models, achieving recall, precision, and F1-score values of approximately 99.32%, 94.35%, and 96.72%, respectively.

These comprehensive evaluation results conclusively affirm the superior performance of our proposed method when compared with previous models for the location/segmentation task, establishing it as a robust and effective approach in the domain of medical image analysis and segmentation.

### Limitations

The proposed method had limitations, including solely relying on using the cross-validation strategy on one public dataset without testing on a separate testing group. This could potentially lead to overfitting and the limited generalizability of the model. Additionally, there was an increased occurrence of false positive segmentation, particularly in areas close to bones, where the grayscale intensity of the image resembled that of intracerebral hemorrhage (ICH) regions, as observed in Figure 8. Another constraint was the difficulty of accurately localizing the ICH regions within the CT scans of two subjects with small intraparenchymal hemorrhage (IPH) regions. Consequently, while the current approach may serve as supportive software for radiologists in ICH segmentation, it has not yet reached a precision level suitable for autonomous segmentation. Future endeavors could involve expanding the dataset by collecting additional CT scans and augmenting the proposed deep neural network (DNN) with a recurrent neural network, such as Long Short-Term Memory (LSTM) networks. This enhancement would address the inter-slice relationships when segmenting ICH regions, potentially improving the model’s performance and precision.

## 5. Conclusions

Intracranial hemorrhages (ICHs) are serious medical injuries that require immediate medical attention, as they can otherwise lead to secondary brain injuries resulting in paralysis or even death. In this paper, we propose a new approach to the segmentation of intracranial hemorrhages based on the data augmentation method. We constructed our segmentation network for intracranial hemorrhages by modifying the conventional U-Net network. We used residual blocks to manipulate the image information provided to the network instead of using conventional convolutional layers. Our approach was validated using a dataset comprising 75 CT scan images out of 82 scans, achieving an IOU score index of 0.807 ± 0.03 on the test set. This result indicates the efficacy of our method in accurately segmenting intracerebral hemorrhages. The proposed method is promising for medical application in the diagnosis of cerebral hemorrhages based on CT scan images. Future work should include tests on more datasets from various populations and scanning devices to verify the solution’s universality and adaptability. Comprehensive comparisons with other state-of-the-art solutions will be a future research topic. To encourage further research and collaboration, we have made our implementation publicly available on our official website [31]. This ensures accessibility for other researchers seeking reference and comparison for their studies.

## Figures and Tables

**Table 1:** Brief description of the dataset used in our experiments.

Number of Patients (Number of CT Scans)	Number of Cases Diagnosed with Intracerebral Hemorrhage	Number of Cases Diagnosed with Skull Fracture	Total Number of Slices	CT Scan’s Average Slices
75	36	22	2814	30

**Table 2:** Dataset after using data augmentation methods.

Method	Subtype					
	Epidural	Intraparenchymal	Intraventricular	Subarachnoid	Subdural	Total
Original	173	73	24	18	56	318
Horizontal flip			24	18		
Vertical flip			24	18		
Increase brightness 10%		73	24	18	56	
Decrease brightness 10%			24	18	56	
Rotation 10 degrees		73	24	18	56	
Rotation −5 degrees			24	18		
Total	173	219	168	126	224	832

**Table 3:** Performance evaluation as a detection framework compares our proposed method with previous detection frameworks with a 10-fold cross-validation procedure (Unit: %).

Method	Recall	Precision	F1-Score
Residual U-Net [19]	98.75 ± 0.20	94.31 ± 8.86	96.42 ± 5.14
FCN-AlexNet [21]	90.81 ± 3.68	86.03 ± 3.77	88.35 ± 3.73
FRCNN [21]	91.40 ± 3.68	93.72 ± 3.80	92.56 ± 3.74
UNet++ [30]	98.69 ± 0.08	92.51 ± 6.38	95.43 ± 5.34
Ours	99.32 ± 0.02	94.35 ± 4.40	96.72 ± 2.46

**Table 4:** Performance evaluation as a segmentation framework comparing our proposed approach with previous detection frameworks with a 10-fold cross-validation procedure. (Unit: %).

Method	IOU Score	Specificity	Accuracy
Residual U-Net [19]	74.56 ± 5.35	98.00 ± 0.71	95.76 ± 0.26
UNet++ [30]	68.04 ± 3.02	96.41 ± 0.89	95.07 ± 0.68
Ours	80.75 ± 3.05	98.17 ± 0.78	96.06 ± 0.04

**Table 5:** Demographic and clinical characteristics of the original dataset. (Unit: %).

Method	IOU-0.9	Specificity	Accuracy
Year > 18	77.30	98.00	95.63
Year < 18	90.48	98.41	96.76
Male	80.00	98.72	95.26
Female	80.75	98.17	96.35

## Data Availability

No new data were created or analyzed in this study. Data sharing is not applicable to this article.

## References

[1] Nolan S. (2005). Traumatic brain injury: A review. Crit. Care Nurs. Q..

[2] Rao V., Lyketsos C. (2000). Neuropsychiatric sequelae of traumatic brain injury. Psychosomatics.

[3] Ayaz H., Izzetoglu M., Izzetoglu K., Onaral B., Dor B.B. (2019). Early diagnosis of traumatic intracranial hematomas. J. Biomed. Opt..

[4] Ho M.-L., Rojas R., Eisenberg R.L. (2012). Cerebral edema. Am. J. Roentgenol..

[5] Rehder D. (2020). Idiopathic intracranial hypertension: A review of clinical syndrome, imaging findings, and treatment. Curr. Probl. Diagn. Radiol..

[6] Fisher C.M. (1995). Brain herniation: A revision of classical concepts. Can. J. Neurol. Sci..

[7] Pan J., Konstas A.-A., Bateman B., Ortolano G.A., Spellman J.P. (2007). Reperfusion injury following cerebral ischemia: Pathophysiology, MR imaging, and potential therapies. Neuroradiology.

[8] Castiglione A., Vijayakumar P., Nappi M., Sadiq S., Umer M. (2021). COVID-19: Automatic detection of the novel coronavirus disease from CT images using an optimized convolutional neural network. IEEE Trans. Ind. Inform..

[9] Vasilakakis M., Iosifidou V., Fragkaki P., Iakovidis D. Bone fracture identification in X-ray images using fuzzy wavelet features. Proceedings of the 19th IEEE International Conference on Bioinformatics and Bioengineering (BIBE).

[10] Nguyen D.T., Pham T.D., Batchuluun G., Yoon H., Park K.R. (2019). Artificial intelligence-based thyroid nodule classification using information from spatial and frequency domains. J. Clin. Med..

[11] Ibrahim W.H., Osman A., Mohamed Y.I. MRI brain image classification using neural networks. Proceedings of the International Conference on Computing, Electrical and Electronics Engineering.

[12] Jacobs B., Beems T., Stulemeijer M., van Vugt A.B., van der Vliet T.M., Borm G.F., Vos P.E. (2010). Outcome prediction in mild traumatic brain injury: Age and clinical variables are stronger predictors than CT abnormalities. J. Neurotrauma.

[13] Litjens G., Kooi T., Bejnordi B.E., Setio A.A.A., Ciompi F., Ghafoorian M., van der Laak J.A.W.M., van Ginneken B., Sánchez C.I. (2017). A survey on deep learning in medical image analysis. Med. Image Anal..

[14] Kuo W., Häne C., Mukherjee P., Malik J., Yuh E.L. (2019). Expert-level detection of acute intracranial hemorrhage on head computed tomography using deep learning. Proc. Natl. Acad. Sci. USA.

[15] Yuan Y., Li Z., Tu W., Zhu Y. (2023). Computed tomography image segmentation of irregular cerebral hemorrhage lesions based on improved U-Net. J. Radiat. Res. Appl. Sci..

[16] Long J., Shelhamer E., Darrell T. Fully convolutional networks for semantic segmentation. Proceedings of the IEEE Conference on Computer Vision and Pattern Recognition.

[17] Van den Heuvel T., van der Eerden A., Manniesing R., Ghafoorian M., Tan T., Andriessen T., Vyvere T.V., Hauwe L.v.D., Romeny B.t.H., Goraj B. (2016). Automated detection of cerebral microbleeds in patients with traumatic brain injury. NeuroImage Clin..

[18] Hssayeni M., Croock M., Salman A., Al-khafaji H., Yahya Z., Ghoraani B. (2020). Computed tomography images for intracranial hemorrhage detection and segmentation. Intracranial Hemorrhage Segmentation Using a Deep Convolutional Model. Data.

[19] Ronneberger O., Fischer P., U-net T.B. Convolutional networks for biomedical image segmentation. Proceedings of the Medical Image Computing and Computer-Assisted Intervention (MICCAI).

[20] Elpeltagy M., Sallam H. (2021). Automatic prediction of COVID-19 from chest images using modified ResNet50. Multimed. Tools Appl..

[21] Yap M.H., Pons G., Marti J., Ganau S., Sentis M., Zwiggelaar R., Davison A.K., Marti R. (2017). Automated breast ultrasound lesions detection using convolutional neural networks. IEEE J. Biomed. Health Inform..

[22] He K., Zhang X., Ren S., Sun J. Deep residual learning for image recognition. Proceedings of the IEEE Conference on Computer Vision and Pattern Recognition.

[23] Yu F., Wang D., Shelhamer E., Darrell T. Deep layer aggregation. Proceedings of the IEEE Conference on Computer Vision and Pattern Recognition.

[24] Hoang Q.T., Pham X.H., Le A.V., Bui T.T. (2023). Artificial Intelligence-Based Breast Nodule Segmentation Using Multi-Scale Images and Convolutional Network. KSII Trans. Internet Inf. Syst..

[25] Malhotra R., Meena S. (2021). Empirical validation of cross-version and 10-fold cross-validation for defect prediction. Proceedings of the 2021 Second International Conference on Electronics and Sustainable Communication Systems (ICESC).

[26] Prabakaran V., Le A.V., Kyaw P.T., Kandasamy P., Paing A., Mohan R.E. (2023). sTetro-D: A deep learning based autonomous descending-stair cleaning robot. Eng. Appl. Artif. Intell..

[27] Gollapudi S., Gollapudi S. (2019). OpenCV with Python. Learn Computer Vision Using OpenCV: With Deep Learning CNNs and RNNs.

[28] Abadi M., Agarwal A., Barham P., Brevdo E., Chen Z., Citro C., Corrado G.S., Davis A., Dean J., Devin M. (2016). Tensorflow: Large-scale machine learning on heterogeneous distributed systems. arXiv.

[29] Liang J. (2020). Image classification based on RESNET. Journal of Physics: Conference Series.

[30] Zhou Z., Siddiquee M.M.R., Tajbakhsh N., Liang J. (2018). UNet++: A Nested U-Net Architecture for Medical Image Segmentation. arXiv.

[31] HYU-ICH-SEGNET http://fme.utehy.edu.vn/AI_lab.

